# Single Femtosecond Laser-Pulse-Induced Superficial Amorphization and Re-Crystallization of Silicon

**DOI:** 10.3390/ma14071651

**Published:** 2021-03-27

**Authors:** Camilo Florian, Daniel Fischer, Katharina Freiberg, Matthias Duwe, Mario Sahre, Stefan Schneider, Andreas Hertwig, Jörg Krüger, Markus Rettenmayr, Uwe Beck, Andreas Undisz, Jörn Bonse

**Affiliations:** 1 Bundesanstalt für Materialforschung und -prüfung (BAM), Unter den Eichen 87, D-12205 Berlin, Germany; camilo.florian@princeton.edu (C.F.); daniel.fischer@rfischergmbh.de (D.F.); mario.sahre@bam.de (M.S.); andreas.hertwig@bam.de (A.H.); joerg.krueger@bam.de (J.K.); uwe.beck@bam.de (U.B.); 2 Princeton Institute for the Science and Technology of Materials (PRISM), Princeton University, 70 Prospect Avenue, Princeton, NJ 08540, USA; 3 Otto-Schott-Institut für Materialforschung (OSIM), Lehrstuhl für Metallische Werkstoffe, Friedrich-Schiller-Universität Jena, D-07743 Jena, Germany; katharina.freiberg@uni-jena.de (K.F.); m.rettenmayr@uni-jena.de (M.R.); 4 Accurion GmbH, Stresemannstraße 30, D-37079 Göttingen, Germany; mdu@accurion.com (M.D.); st@accurion.com (S.S.); 5 Institut für Werkstoffwissenschaft und Werkstofftechnik (IWW), Technische Universität Chemnitz, Erfenschlager Straße 73, D-09125 Chemnitz, Germany; andreas.undisz@mb.tu-chemnitz.de

**Keywords:** femtosecond laser, silicon, amorphization, crystallization, spectroscopic imaging ellipsometry, transmission electron microscopy, atomic force microscopy

## Abstract

Superficial amorphization and re-crystallization of silicon in <111> and <100> orientation after irradiation by femtosecond laser pulses (790 nm, 30 fs) are studied using optical imaging and transmission electron microscopy. Spectroscopic imaging ellipsometry (SIE) allows fast data acquisition at multiple wavelengths and provides experimental data for calculating nanometric amorphous layer thickness profiles with micrometric lateral resolution based on a thin-film layer model. For a radially Gaussian laser beam and at moderate peak fluences above the melting and below the ablation thresholds, laterally parabolic amorphous layer profiles with maximum thicknesses of several tens of nanometers were quantitatively attained. The accuracy of the calculations is verified experimentally by high-resolution transmission electron microscopy (HRTEM) and energy dispersive X-ray spectroscopy (STEM-EDX). Along with topographic information obtained by atomic force microscopy (AFM), a comprehensive picture of the superficial re-solidification of silicon after local melting by femtosecond laser pulses is drawn.

## 1. Introduction

Through the rapid recent developments in laser technology in combination with advanced optical beam management strategies, laser processing has nowadays entered many industrial production processes [1,2]. In most cases, the processing relies on the laser-induced removal of material, i.e., on laser ablation [3]—a process that exhibits a sharply defined threshold of the fluence (areal energy density) of the incident laser radiation. However, below the ablation threshold fluence, other material specific phenomena may occur that also exhibit a distinct threshold fluence, e.g., structural or chemical changes triggered by effects such as melting [3], oxidation [4], re-crystallization [5,6], etc. Some of these non-ablative phenomena are widely explored and already used in applications, e.g., in optical data storage [7,8,9,10].

Given its technological relevance, silicon is the material that is studied best with regard to the interaction with intense laser radiation. Experimentally, it can be manufactured in a well-controlled manner and theoretically it is already well understood [11]. Observed effects are usually interpreted as laser-induced phase transitions and have very actively been investigated over the last five decades while exploring the possibility of removing defects from crystalline semiconductor surfaces through laser irradiation (a process referred to as laser annealing) [3,12,13,14].

Studying interactions of intense ultrashort (fs-ps) laser pulses with semiconductors gave rise to exploring the intriguing effect of non-thermal melting, where a strong optical excitation of the electronic system of the solid may lead to a destabilization of the bonding lattice structure on an ultrafast (sub-ps) timescale [15,16,17,18,19,20,21,22,23,24]. For silicon, this effect typically manifests at conduction band carrier densities >10^22^ cm^−3^ (corresponding to ~10% of the total valence band population) [16,25] at laser fluences exceeding the ablation threshold [26]. In contrast, at lower degrees of optical excitation, thermal melting relies on the process of electron–phonon scattering and, thus, occurs on longer timescales of picoseconds after the impact of the laser pulse. Laser-induced thermal melting of bulk semiconductors usually manifests via heterogeneous nucleation as an interfacial melt-in, followed by interfacial re-solidification processes [27,28,29], while locally converting (releasing) the latent heat of fusion. Driven by the gradual increase of undercooling of the superficial melt pool via heat conduction into the bulk material, the velocity of the solid/liquid interface may accelerate toward the end of the re-solidification process [27]. Depending on the velocity of the re-solidification front, the quenched material can re-solidify either epitaxially as a single-crystal, or it can turn into a less ordered poly-crystalline or even into an amorphous state since the atoms have not enough time to form an ordered lattice [14]. For semiconductors, the critical interfacial velocity (*v*_crit_) for solidification as amorphous material typically lies between a few m/s and some tens of m/s, and crucially depends on the interfacial lattice orientation of the crystalline substrate [14,30] and the type of the semiconductor [14,23,31,32,33,34]. Note that the energy deposition depth of ultrashort laser pulse irradiation may significantly differ for semiconductors and metals and will lead to different outcomes regarding local melting and solidification. Therefore, kinetic aspects are linked to the specific laser irradiation conditions. Here, the reader is referred to pertinent literature, including thermodynamic and atomistic numerical studies [27,29,32,34,35,36].

Upon irradiation of semiconductors with ultrashort (fs-ps) laser pulses, amorphous surface layers with a thickness of some tens of nanometers were reported, as determined by analytical techniques such as transmission electron microscopy (TEM), electron backscatter diffraction (EBSD) analyses, micro-Raman spectroscopy (µ-RS), confocal scanning laser microscopy (CSLM), real-time reflectivity measurements (RTR), and fs-time-resolved microscopy (fs-TRM) [33,34,36,37,38,39,40,41,42,43,44,45,46,47]. The specific interest of such a contact-less surface modification technology is based on the remarkably different structural, electrical and optical properties of the amorphous and the crystalline silicon phases that enable applications in electronics and photonics and manifest through alterations of the local chemical etching rate, electric conductivity, or the high refractive indices at telecom wavelengths [48,49].

In a previous publication [42], a non-destructive all-optical method to reconstruct the radial depth profile of the amorphous layer was proposed that is inferred upon single-pulse irradiation of single-crystalline <111> silicon with a spatially Gaussian fs-laser beam. It relies on CSLM measurements of the surface reflectivity across a laser-amorphized spot along with a thin-film optical model considering all Fresnel reflections of a system consisting of an amorphous silicon layer (a-Si) of varying thickness, covered by a native oxide (~3 nm) and sitting on a substrate of single-crystalline silicon (c-Si) [40,42]. A laterally parabolic a-Si layer thickness distribution with a maximum vertical extent of ~60 nm was retrieved from the reflectivity measurements for a surface spot amorphized by a single 130 fs Ti:Sapphire laser pulse at a peak fluence of *ϕ*_0_ = 0.42 J/cm^2^. A direct measurement of the amorphous layer thickness by chemical etching was proposed but could not be performed at that time.

In this work, we extend the previous study [42] by employing spectroscopic imaging ellipsometry (SIE) for a fast non-destructive quantitative determination of the lateral thickness gradient of the superficial amorphous layer induced by single spatially Gaussian-shaped 30 fs laser pulses of varying energies (peak fluences) on two different silicon wafers with varying crystal lattice orientation. Taking advantage of the spectroscopic ellipsometric approach along with the well-known radial beam profile, a model of the fluence dependence of the laser-induced amorphization is determined with unprecedented precision and experimentally verified by cross-sectional high-resolution transmission electron microscopy (HRTEM) and energy dispersive X-ray spectroscopy (STEM-EDX).

## 2. Materials and Methods

Two single-crystalline silicon (c-Si) wafers with different orientation (Werk für Fernsehelektronik, Berlin, Germany) were chosen as target materials, specifically Czochralsky-grown n-type Si<111> (electrical resistivity 1.3 × 10^−2^–1.9 × 10^−2^ Ω.cm) and p-type Si<100> (electrical resistivity 1.2 × 10^−1^–2 × 10^−1^ Ω.cm) wafers, both with a thickness of ~0.4 mm and a diameter of two inches. On the polished silicon samples, typically a native oxide layer of about 1 nm to 3 nm thickness is present due to the exposure to ambient air.

The silicon wafers were irradiated at ambient conditions by near-infrared fs-laser pulses generated by a commercial Ti:Sapphire multi-pass amplifier system (Compact Pro, Femtolasers, Vienna, Austria). The laser system emitted linearly polarized laser pulses of τ~30 fs duration at a center wavelength of λ~790 nm. The laser pulse energy was measured by a pyroelectric detector. The wafers were mounted on a motorized *x-y-z*-translation stage and positioned perpendicular to the direction of the laser beam (*z*). The fs-laser beam was focused by a spherical dielectric mirror (focal length *f* = 500 mm) to the front side of the samples, resulting in a Gaussian (1/e^2^) radial spot diameter of 2*w*_0_ = 112.6 µm, as determined by the method of Liu [50]. By means of a half-wave plate in front of the compressor unit of the laser amplifier, the laser pulse energy *E*_p_ of the focused laser beam was varied between 10 µJ and 40 µJ, corresponding to incident peak fluences *ϕ*_0_ = 2*E*_p_/(π*w*_0_^2^) in front of the samples between 0.20 J/cm^2^ and 0.80 J/cm^2^. For a given laser pulse energy, the radial fluence profile *ϕ*(*r*) incident to a surface spot can then be calculated via

(1) $$\phi \left(r\right)=\frac{2E_{p}}{\pi w_{0}^{2}}e^{-2{\left(\frac{r}{w_{0}}\right)}^{2}}$$

**Figure 1:**
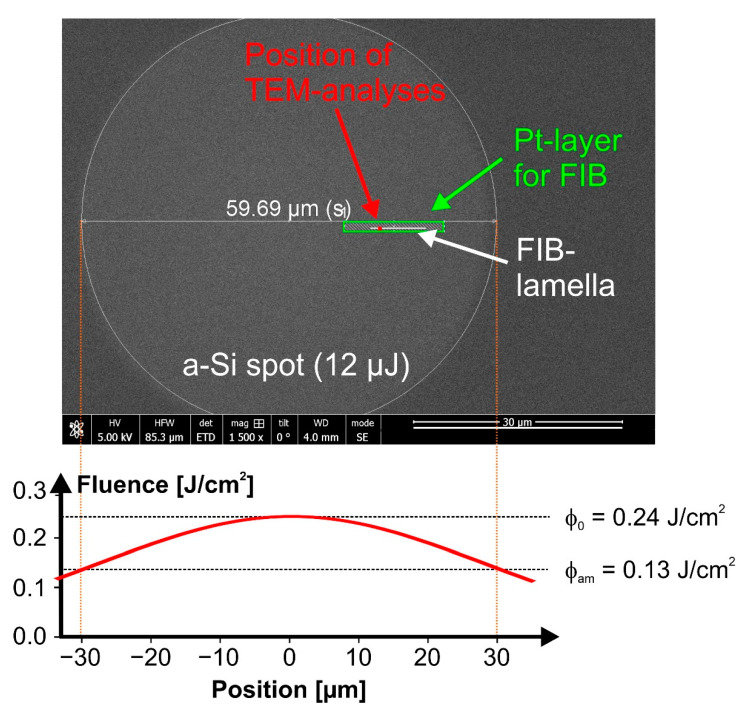
Top-view SEM image of an amorphous (a-Si) spot created by a single fs-laser pulse [λ~790 nm, τ = 30 fs, *E*_p_ = 12 µJ/*ϕ*_0_ = 0.24 J/cm^2^] on the surface of a Si<111> wafer. Its diameter of 59.69 µm is indicated as an additional marking in white. The plot in the lower part visualizes the corresponding radially Gaussian laser beam profile through the center of the laser beam along with the peak fluence (*ϕ*_0_) and amorphization threshold fluence (*ϕ*_am_). The rectangular green box indicates the region of the post-irradiation deposition of a Pt-layer prior to the focused ion beam (FIB) preparation process. The horizontal white line within that box marks the orientation of the FIB lamella, while the red square indicates the position of transmission electron microscopy (TEM)/scanning transmission electron microscopy (STEM) imaging.

As an example, such a radial Gaussian fluence profile is quantitatively visualized as a red curve in the lower part of Figure 1 for a laser pulse of *E*_p_ = 12 µJ, resulting in a peak fluence value of (*ϕ*_0_ = 0.24 J/cm^2^).

During laser processing, the sample translation stages were simultaneously moved at constant speeds in both the *x*- and the *y*-directions, while continuously irradiating the wafers at 1 kHz pulse repetition rate. In this way, for each selected laser pulse energy (peak fluence) a set of several almost identical laser irradiation spots were generated, laterally separated at the sample surfaces, and were available for further post-irradiation characterizations.

The irradiated wafer surface regions were inspected by an optical microscope in brightfield imaging mode (Eclipse L200, Nikon, Tokyo, Japan) using a white light halogen lamp for illumination along with a 10× microscope objective.

Surface topographies of all laser-irradiated spots were acquired by atomic force microscopy (AFM, Dimension 3100, Digital Instruments, Santa Barbara, CA, USA) in tapping mode using silicon cantilever probes with a nominal tip radius of 10 nm under ambient laboratory conditions. The corresponding images of 100 × 100 µm^2^ size contain 512 data points in *x*-, and 256 lines in *y*-direction, with a nominal resolution in *z* of 70 pm (4.6 µm/16 bit in the full *z*-scale range). The data are displayed as two-dimensional color maps of the surface topography, using a common color scale for encoding topographic height (*z*-) variations ranging between −30 nm and +60 nm, with the original surface plane located at *z* = 0.

Further optical characterization of all laser-irradiated spots on Si<100> and Si<111> was performed using a Nanofilm_EP3 imaging ellipsometer (Accurion GmbH, Göttingen, Germany) equipped with a Nikon 10× achromatic long working-distance microscope objective (NA 0.21), operated at an angle of incidence (AOI) of 45° with a continuous wave (cw) diode laser emitting at 637 nm wavelength as illumination source. The measurement applied a polarizer–analyzer nulling scheme and directly yielded two-dimensional micrographs of the ellipsometric quantities Ψ and Δ (called Ψ-Δ-maps hereafter). By definition, the value of tan(Ψ) denotes the amplitude ratio of the complex reflection coefficients for p- and s-polarized light waves. Δ equals the sample-induced phase-shift between p- and s-polarized waves and is very sensitive to variations of the (optical) layer thickness of thin surface layers.

For quantification of the lateral amorphous layer profiles of selected fs-laser-irradiated spots in the form of thickness maps, a Nanofilm_EP4 spectroscopic imaging ellipsometer (Accurion GmbH, Göttingen, Germany) was applied to acquire hyperspectral Ψ-Δ-maps (wavelength of illumination ranging from 370 to 950 nm in 10 nm steps) using a laser-stabilized Xenon broadband light source and a grating monochromator for the sample illumination (bandwidths 4.6 nm to 5.2 nm). The measurements were performed using the instrument’s rotating compensator mode in combination with achromatic long working-distance microscope objectives (Nikon 20× NA 0.35 and Nikon 10× NA 0.21) at AOIs of 45° (20×) and 55° (10×) for the Si<100> and Si<111> samples, respectively. The corresponding field of view (FOV) sizes were approximately 500 × 500 μm^2^ (10×) and 250 × 250 μm^2^ (20×) with effective image pixel sizes of 0.586 × 0.586 µm^2^ and 0.362 × 0.362 µm^2^, respectively. At the given conditions, the imaging resolution is better than 2 µm. Details on the ellipsometry instrumentation, the setup and the modes of operation may be found elsewhere [51,52,53,54].

Top-view scanning electron microscopy (SEM) allowed to locate the laser-irradiated spots at the surface due to the altered morphology or electrical conductivity of the smooth superficially amorphized material. Cross-sections perpendicular to the surface were characterized by HRTEM and STEM-EDX combined with an energy dispersive X-ray spectroscopy system at a spot on the Si<111> wafer irradiated at *E*_p_ = 12 µJ (*ϕ*_0_ = 0.24 J/cm^2^). Surface-preserving sample preparation for TEM was carried out using focused ion beam (FIB) milling and an in-situ lift-out technique [55]. The preparation of the TEM lamella started by the deposition of a protective Pt capping layer at the region of interest (ROI). Figure 1 shows a schematic of a top-view scanning electron microscopy (SEM) image of the selected laser-amorphized spot along with the off-centered ROI marked by a green hatched box. The lamella was investigated using a NEOARM 200F (JEOL, Freising, Germany) analytical transmission electron microscope operating at 200 kV electron acceleration voltage. The device was equipped with a cold field emission gun, 4k × 4k CCD-camera (OneView, Gatan Inc., Pleasanton, CA, USA) and a windowless double solid-state detector with a total detector area of 200 mm^2^ (Centurio, JEOL, Freising, Germany) for EDX. The orientation of the lamella and the location for high-resolution imaging are additionally indicated by the horizontal white line and the red square symbol in the green box in Figure 1.

## 3. Results

**Figure 2:**
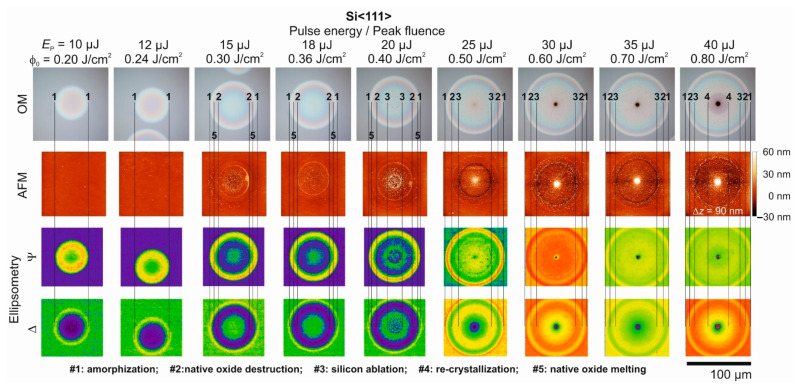
Comparison of brightfield optical microscopy (OM) images, atomic force microscopy (AFM) images, and corresponding spatial maps of the ellipsometric quantities Ψ and Δ (taken at 637 nm wavelength) for a series of nine single fs-laser [λ~790 nm, τ = 30 fs] pulse-irradiated spots on a Si<111> wafer. The fs-laser pulse energy *E*_p_ varies between 10 µJ and 40 µJ among the spots from left to right, corresponding to peak laser fluences between *ϕ*_0_ = 0.20 and 0.80 J/cm^2^. The AFM images are provided in a common color scale, encoding height variations Δ*z* of −30 to +60 nm. The vertical (numbered) line sets mark the extent of characteristic surface features (for details see the text at the bottom of the figure). A common lateral scale bar is provided in the lower right corner.

Figure 2 shows a compilation of images of a series of nine laser-irradiated spots at the Si<111> wafer generated by single fs-laser pulses of different laser pulse energies/peak fluences (as indicated at the top). The upper image row provides optical micrographs, the second row the corresponding atomic force micrographs. The two lower rows display maps of the ellipsometric quantities Ψ and Δ obtained from imaging ellipsometry (IE, Nanofilm_EP3, Accurion GmbH, Göttingen, Germany) for the visualization of changes of the polarization state of the probing 637 nm radiation through the laser-induced surface modifications. For the most sensitive visualization of laser-induced alterations, the ellipsometry maps are displayed in a multi-color scheme with individually optimized color ranges.

Several characteristic annular structures can be recognized in the series of laser-irradiated surface spots displayed in Figure 2. In a previous work [40] employing single Ti:Sapphire fs-laser pulses (800 nm, 130 fs) these annuli were already associated with physical processes of laser-induced melting of silicon with subsequent amorphization (outermost modification; line set #1), the local destruction of the native oxide layer (line set #2), ablation of silicon (line set #3), and the onset of (poly-crystalline) re-crystallization in the center of the spot exposed to the highest laser pulse energies (line set #4). The latter can be explained as follows: the interfacial velocities and lifetimes of the melt pool are strongly affected by the temperature gradients and the amount of latent heat stored in the liquid layer of transiently varying thickness (with a maximum extent initially imposed by the peak laser fluence). The release of latent heat of crystallization (enthalpy of fusion) can retard the re-solidification, particularly in the central region of the irradiated spot, where the lifetime of the melt layer is the longest. Hence, at large laser pulse energies (peak fluences), re-crystallization is possible [5]. For a scheme visualizing the different processes, the reader is referred to the graphical abstract of this article. The origin of the modification of line set #5 in an intermediate range of local fluences is not fully clear yet; supposedly, it is associated with a local non-ablative modification of the native oxide layer, e.g., via melting and effects of further oxidation. The assignments of the origin of the features associated with the line sets #2 and #5 to the native oxide layer are supported by some thermodynamic arguments, i.e., that silicon melts at a lower temperature than amorphous silicon dioxide (*T*_m_(c-Si)~1685 K < *T*_m_(a-SiO_2_)~1983 K, [56]), while the evaporation temperature [*T*_ev_(a-SiO_2_)~2503 K] of amorphous silicon dioxide lies between the melting temperature and the evaporation temperature *T*_ev_(c-Si)~3510 K of crystalline silicon [3].

The laser pulse energy/peak fluence dependence of the diameters of the distinct features marked by the different line sets was analyzed in detail by means of the method proposed by Liu [50], providing characteristic single-pulse threshold fluences of *ϕ*_am_(Si<111>) = 0.13 J/cm^2^ for melting with subsequent amorphization (from line set #1), *ϕ*_annu_(Si<111>) = 0.26 J/cm^2^ for the destruction of the native oxide layer (from line set #2), *ϕ*_abl_(Si<111>) = 0.39 J/cm^2^ for ablation (from line set #3). Additionally, a value of *ϕ*_cr_(Si<111>) = 0.68 J/cm^2^ was estimated from the diameter of the re-crystallization feature of the laser spot irradiated at 40 µJ (*ϕ*_0_ = 0.80 J/cm^2^) along with its known radial fluence profile (Equation (1)). These threshold values of melting and ablation are somewhat smaller than the values previously reported in [40] since a 4 to 5 times shorter pulse duration is employed here. For the amorphization threshold fluence, this is well supported by the theoretical work of Rämer et al. [57] providing a melting threshold of 0.125 J/cm^2^ for silicon irradiated by single 50 fs laser pulses at 800 nm wavelength. In contrast, the threshold of re-crystallization is particularly increased since the Gaussian beam diameter is larger by a factor of ~2 here, resulting in a larger melt pool that increases the total amount of latent heat stored and released during the re-crystallization.

The most striking observation in the OM images is a significant increase of the surface reflectivity within the laser-modified spots on Si<111> (line set #1 in Figure 2). Previous micro-Raman spectroscopy results already revealed the presence of amorphous material in these regions [40]. The significant changes of the refractive index (*n*) and the extinction coefficient (*k*) of amorphous silicon can then explain the increase of the surface reflectivity. The color changes within the amorphous regions point toward interference effects through a wavelength dependence of the optical constants (*n*, *k*) along with a laterally varying thickness of the superficial semi-transparent amorphous layer upon the white light illumination employed in brightfield optical microscopy. As expected, in regions outside of the destruction of the native oxide layer (line set #2) no surface topographic changes were observed by AFM (see the spots irradiated at *E*_p_ = 10 µJ (*ϕ*_0_ = 0.20 J/cm^2^) and *E*_p_ = 12 µJ (*ϕ*_0_ = 0.24 J/cm^2^)). The effect manifests for all pulse energies larger than *E*_p_ = 15 µJ (*ϕ*_0_ = 0.30 J/cm^2^) as a very shallow and fine annular rim (height ~15 nm ± 5 nm, width < 2 µm) in the AFM images that was not resolved by OM here. Inside those regions, a small increase of the surface roughness can be observed in the AFM images, resulting from residuals of the laser-induced decomposition of the native oxide layer. For laser pulse energies exceeding *E*_p_ = 20 µJ (*ϕ*_0_ = 0.40 J/cm^2^), additionally the ablative removal of silicon from the surface can be seen (bordered by line set #3), resulting in some few tens of nanometers deep ablation craters along with a further increased surface roughness. Interestingly, at laser pulse energies above *E*_p_ = 25 µJ (*ϕ*_0_ = 0.50 J/cm^2^) a characteristic protrusion even exceeding the original surface plane by heights of up to ~60 nm manifests in the center of the ablation craters. For the highest laser pulse energies, this central hillock becomes visible as dark spot in the center of the optical micrographs. Supposedly, this central protrusion is caused by radial transport of molten silicon toward the center of the spots caused by the volume expansion of liquid silicon by up to ~9% during the re-solidification [40,58]. Note that at room temperature the mass density/specific volume of amorphous silicon is ~1.8% smaller/larger than that of single-crystalline silicon [59]. In such a scenario, the laser-induced melt pool solidifies from its bottom toward the surface and simultaneously from its outer border toward the center of the irradiated spot. The material expansion of silicon upon re-solidification then “squeezes” the still molten silicon to the center, where the solidification process finally terminates. Here, surface oxidation effects may be strongest and additionally affect the surface reflectivity. Melt flow caused by the thermocapillary (Marangoni) effect can be ruled out here as, for a spatially Gaussian laser beam, it would result in a radially outward directed displacement of the silicon [60,61].

The ellipsometric maps of Ψ and Δ visualize most of the mentioned annular features and are able to expose even the subtle surface features detected by AFM that are not resolved in OM here. Both ellipsometric quantities are very sensitive to changes of the optical properties of the silicon material, manifesting either via structural material changes such as amorphization or due to thickness variations of the surface layers. Even the regions associated with the destruction of the native oxide layer (bordered by line set #2) can be distinguished as a step-like local jump in Ψ for laser pulse energies between 15 µJ (*ϕ*_0_ = 0.30 J/cm^2^) and 18 µJ (*ϕ*_0_ = 0.36 J/cm^2^), before stronger changes caused by ablation and re-solidification dominate the ellipsometry maps.

**Figure 3:**
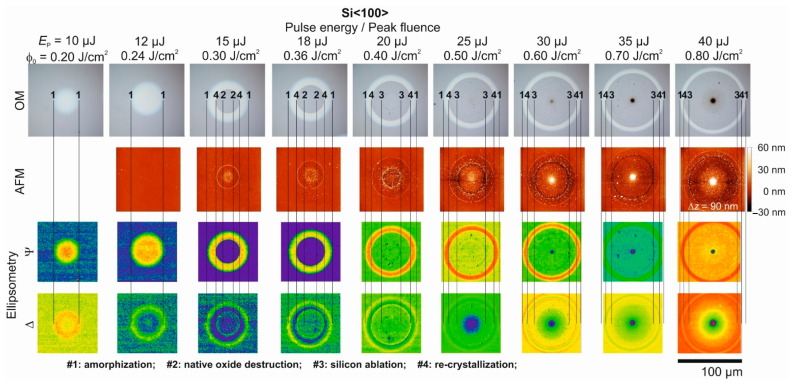
Comparison of brightfield optical microscopy (OM) images, atomic force microscopy (AFM) images, and corresponding spatial maps of the ellipsometric quantities Ψ and Δ (taken at 637 nm wavelength) for a series of nine single fs-laser (λ~790 nm, τ = 30 fs) pulse-irradiated spots on a Si<100> wafer. The fs-laser pulse energy *E*_p_ varies between 10 µJ and 40 µJ among the spots from left to right, corresponding to peak laser fluences between *ϕ*_0_ = 0.20 and 0.80 J/cm^2^. The AFM images are provided in a common color scale, encoding height variations Δ*z* of −30 to +60 nm. The vertical (numbered) line sets mark the extent of characteristic surface features (for details see the text at the bottom of the figure). A common lateral scale bar is provided in the lower right corner.

Figure 3 displays an analogous collage of identical experimental data for the single fs-laser pulse irradiation of the Si<100> wafer material. The most striking differences to the data previously shown in Figure 2 are the somewhat smaller diameters of the laser-modified spots and, for *E*_p_ ≥ 15 µJ (*ϕ*_0_ ≥ 0.30 J/cm^2^), the occurrence of a central disc-shaped area having the same optical properties as the non-irradiated wafer material in the surrounding of the spots (see the OM and ellipsometric images). This central disc is associated with the process of re-solidification that can occur either in a single- or in a poly-crystalline state [40,47]. It has a reduced threshold value of *ϕ*_cr_ (Si<100>) = 0.25 J/cm^2^ here for the Si<100> compared to Si<111>, fully in line with the results reported by Cullis et al. [14] for the ns-laser irradiation and by Merkle et al. [38] for the ps-laser irradiation of silicon wafers with different interfacial crystal orientations; depending on the orientation of the crystalline lattice, values of *v*_crit_(Si<111>) ~11 m/s and *v*_crit_(Si<100>) ~15 m/s were determined for the critical interfacial velocity upon ns-laser irradiation [14]. Bucksbaum et al. [32] reported for UV ps-laser irradiation a saturation value of the interfacial velocity of ~25 m/s being widely independent of the peak laser fluences. For pulse energies of *E*_p_ = 15 µJ (*ϕ*_0_ = 0.30 J/cm^2^) and *E*_p_ = 18 µJ (*ϕ*_0_ = 0.60 J/cm^2^) the AFM images indicate the local decomposition of the native silicon oxide layer in the center of the irradiated spots, resulting in an increased surface roughness that manifests also as a slightly elevated (averaged) surface topography. Since OM and SIE mainly image the electronic configuration of the inspected material and both do not have the necessary spatial resolution, these nanometric topography changes are not visible in the corresponding OM and SIE micrographs here. At laser pulse energies exceeding *E*_p_ = 20 µJ (*ϕ*_0_ = 0.40 J/cm^2^) the ablation of silicon becomes visible as a fine rim bordering the ablation craters in the OM, AFM, and ellipsometry micrographs of Figure 3.

### 3.1. Layer Thickness Analysis by Spectroscopic Imaging Ellipsometry (SIE)

For a quantification of the thickness of the amorphous layer profiles, additional multi-wavelength measurements were performed by SIE (Nanofilm_EP4). In order to avoid the complexity imposed by melt displacement, ablation, or the laser-induced modification/destruction of the native oxide layer, this set of SIE measurements was restricted to surface spots on both silicon wafer materials generated at the lowest two laser pulse energies (peak fluences) of *E*_p_ = 10 µJ (*ϕ*_0_ = 0.20 J/cm^2^) and 12 µJ (*ϕ*_0_ = 0.24 J/cm^2^), respectively. These laser irradiation conditions cause laser-induced amorphization on both samples. Each pixel of the acquired hyperspectral Ψ–Δ maps contains an ellipsometric spectrum, i.e., the measured values of Ψ and Δ for 59 different wavelengths of the probing beam, equidistantly spaced in the range from 370 to 950 nm. Pixel by pixel, each of these spectra was then fitted by a computational thin-film multi-layer model using the 2 × 2 transfer matrix calculus for stratified isotropic media (see, for example, [62]) and a least-squares non-linear regression algorithm (Levenberg–Marquardt) as provided by the *Accurion EP4Model* software (Vers. 19.8.3, Accurion GmbH, Göttingen, Germany). The approach assumed a two-layer model (substrate/layer 1/layer 2/ambient) consisting of c-Si (bulk)/a-Si(*d*_am_)/a-SiO_2_(*d*_ox_)/air. The thickness of the amorphous silicon layer (*d*_am_) and that of the covering amorphous silica layer (*d*_ox_) were taken as fit parameters, while the wavelength-dependent optical constants of all different materials were taken from a material database originally provided by the former company Sopra S.A. (France) [63]. Additionally, an offset of the local angle of incidence (AOI) was used as fit parameter to consider changes in the direction of the probing light by the buried a-Si/c-Si interface. Interfacial roughness effects were neglected in the ellipsometric modeling as inferred from the previous AFM measurements. As a result of the fit procedure, the single-pixel values of the fit parameters were converted into parameter maps, i.e., maps of the thickness for the amorphous silicon and silica layers.

**Figure 4:**
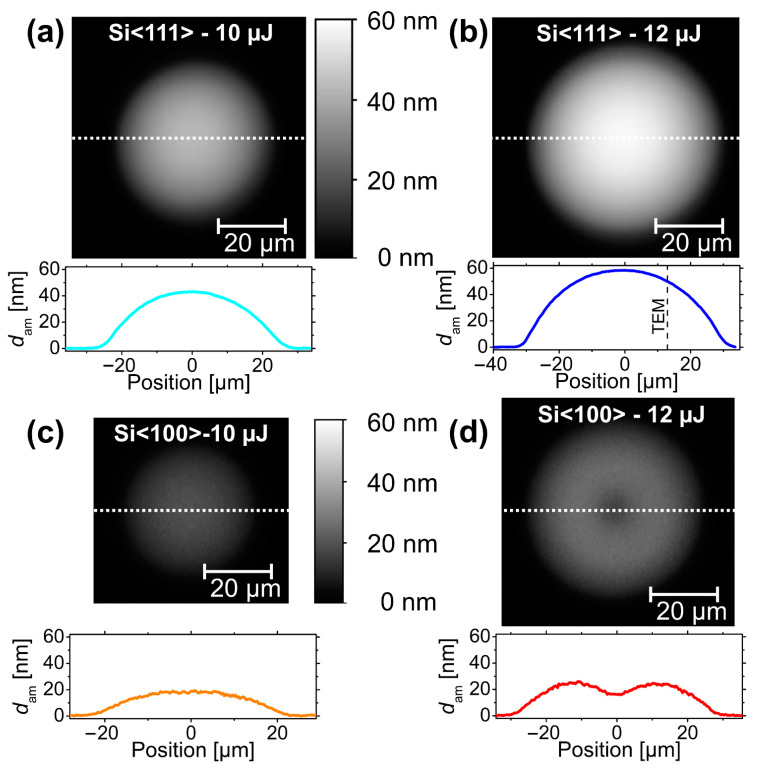
Maps of the amorphous silicon layer thickness (*d*_am_) as determined by spectroscopic imaging ellipsometry (SIE) at four different fs-laser-irradiated spots on Si<111> (**a**,**b**) and Si<100> (**c**,**d**) at two laser pulse energies of 10 µJ (*ϕ*_0_ = 0.20 J/cm^2^; (**a**,**c**)) or 12 µJ (*ϕ*_0_ = 0.24 J/cm^2^; (**b**,**d**)). The maps have a common scale bar provided in (**a**,**c**) and are encoded by a joint linear grayscale that indicates the thickness. Below each map, a corresponding horizontal cross-section (location indicated by a white dashed line) of the a-Si layer thickness is drawn. The vertical black dashed line in (**b**) indicates the position of the TEM/STEM measurements as marked by the red square in Figure 1.

Figure 4 compares the results of the SIE-based evaluation of the amorphous silicon layer thickness *d*_am_(*x*,*y*) for the four selected fs-laser-irradiated spots at the two laser pulse energies and for the two different wafer materials. The images share common lateral scale bars. The amorphous silicon layer thickness *d*_am_ is encoded in a joint linear grayscale with brighter values representing larger thickness values up to 60 nm. The enhanced tendency for laser-induced amorphization for the <111> crystal orientation manifests in larger amorphized spot areas and significantly larger amorphous layer thicknesses when compared to the <100> crystal orientation (compare Figure 4a,b with Figure 4c,d). Moreover, for the spot irradiated at *E*_p_ = 12 µJ (*ϕ*_0_ = 0.24 J/cm^2^) at the Si<100> wafer even a local minimum of *d*_am_ can be seen in the center (Figure 4d) as a precursor to the re-crystallization occurring at somewhat larger peak fluences exceeding *ϕ*_cr_(Si<100>) = 0.25 J/cm^2^.

**Figure 5:**
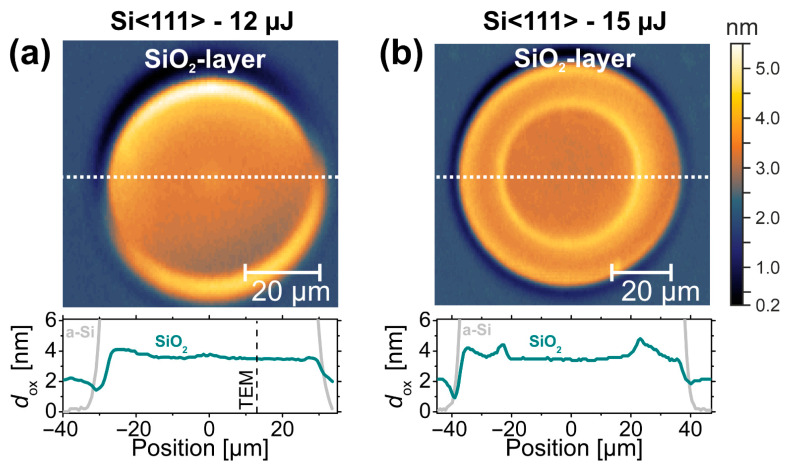
Top: maps of the silicon oxide layer thickness (*d*_ox_) as determined by SIE at the surface spots irradiated on Si<111> by a single laser pulse of (**a**) 12 µJ (*ϕ*_0_ = 0.24 J/cm^2^) and (**b**) 15 µJ (*ϕ*_0_ = 0.30 J/cm^2^); bottom: horizontal cross-sections (location indicated by a white dashed line) of the oxide (SiO_2_) layer thickness (dark cyan curves) along with the data of the a-Si layer thickness (gray curves) for comparison. The vertical black dashed line in (**a**) indicates the position of the TEM/STEM measurements as marked by the red square in Figure 1. The thickness maps share a common color scale provided in (**b**). Note the different image sizes.

The simultaneous fit of the amorphous silica (SiO_2_) cover layer yielded an almost constant thickness of *d*_ox_~3 nm to 5 nm inside and ~2 nm to 3 nm outside the laser-amorphized regions for each of the examined SIE maps. This is exemplified in Figure 5 visualizing the modeled spatial variation of the silicon oxide layer thickness for spots on Si<111> irradiated by single pulses of *E*_p_ = 12 µJ/*ϕ*_0_ = 0.24 J/cm^2^ (a, left) and *E*_p_ = 15 µJ/*ϕ*_0_ = 0.30 J/cm^2^ (b, right).

The ring-shaped local maxima/minima in the upper and lower parts of the two maps arise from the ellipsometric imaging geometry performed at non-normal incidence here (AOI = 55°). In view of a typical native oxide layer thickness, the fitted values of *d*_ox_~2 nm outside the laser spot appear reasonable. Comparing the cross-sections visualized by the dark cyan (SiO_2_) and gray (a-Si) curves assembled in the bottom parts of Figure 5a,b indicates that the fs-laser-induced amorphization is additionally accompanied by a local increase of the thickness of the covering oxide layer of ~2 nm here. At the peak fluence *ϕ*_0_ = 0.24 J/cm^2^ below the threshold of the laser-induced destruction of the native oxide layer (*ϕ*_annu_ = 0.26 J/cm^2^), a step-like oxide layer profile can be seen in Figure 5a. Particularly at the location of the TEM/STEM analyses (marked by the black dashed vertical line), the SIE-based modeling predicts a thickness of ~3.5 nm. At the increased peak fluence *ϕ*_0_ = 0.30 J/cm^2^, i.e., above *ϕ*_annu_, a central disc of slightly reduced oxide layer thickness is additionally visible in Figure 5b.

These observations of the oxide layer profiles further support the scenario of the local destruction of the native oxide layer at peak fluences *ϕ*_0_ ≥ *ϕ*_annu_. Since the wafer surface is left at high temperatures *T* > *T*_ev_(a-SiO_2_) ~2503 K in ambient air after the laser irradiation, the laser-ablated silicon oxide re-grows in the center of the irradiated spot (*ϕ*_abl_ > *ϕ* ≥ *ϕ*_annu_) at a transiently increased rate compared to the native oxide of the non-irradiated wafer. In contrast, in the outer annular region, where *ϕ*_am_ ≤ *ϕ* < *ϕ*_annu_, the laser-induced heating of the silicon increased the thickness of the covering oxide layer by ~2 nm to 3 nm here. Note that the local maxima of *d*_ox_ observed in the bottom part of Figure 5b at the positions where *ϕ*_annu_ is locally reached (±22 µm in Figure 5b) are additionally affected by optical scattering effects imposed by the fine topographic rim previously seen in the AFM/OM data of Figure 2.

### 3.2. Layer Analysis by Transmission Electron Microscopy (TEM)

**Figure 6:**
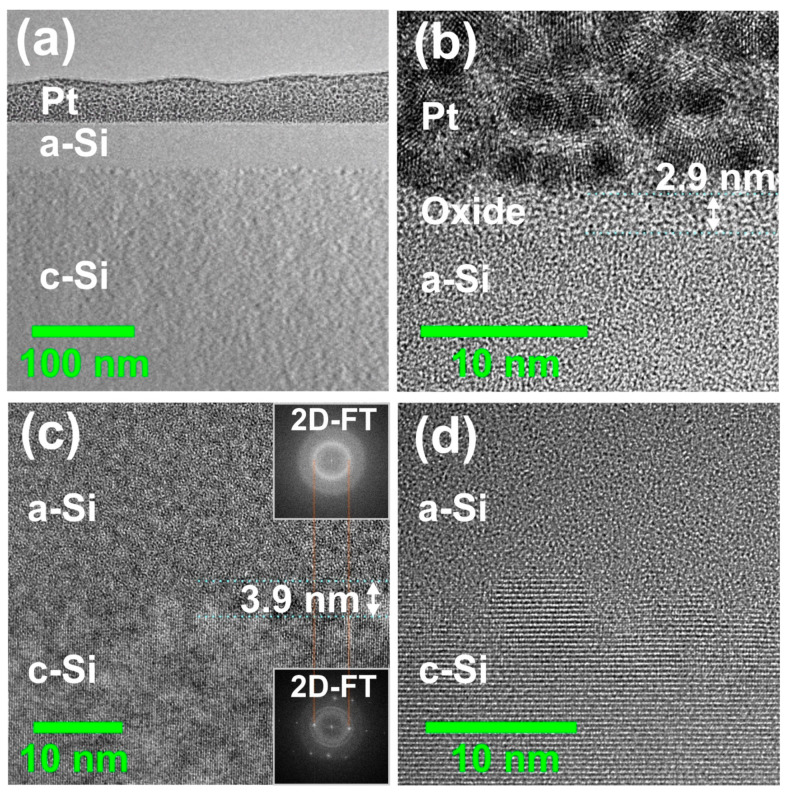
TEM images of an fs-laser-amorphized spot in cross-section (*E*_p_ = 12 µJ/*ϕ*_0_ = 0.24 J/cm^2^) on c-Si<111> taken at the position marked by the red square in Figure 1. (**a**) Overview; (**b**) high-resolution image of native oxide layer between the Pt capping layer and the a-Si layer; (**c**,**d**) high-resolution images of the a-Si/c-Si interface. Note the different magnifications. The insets in (**c**) are two-dimensional fast Fourier transforms (2D-FTs) of high-resolution transmission electron microscopy (HRTEM) images of the a-Si and c-Si regions, respectively.

In order to verify assumptions made in the SIE data modeling and to verify the corresponding amorphous layer thickness results experimentally, cross-sectional TEM/STEM analyses were performed on the spot with the largest a-Si thickness, i.e., for Si<111> irradiated at *E*_p_ = 12 µJ (*ϕ*_0_ = 0.24 J/cm^2^). Figure 6a presents a TEM cross-section through the lamella prepared by FIB at the position marked by the red square in Figure 1. Below the poly-crystalline Pt protection layer, an amorphous a-Si layer of almost constant thickness (*d*_am_~42 nm to 44 nm) was measured at this specific position on top of the crystalline silicon material of the substrate (c-Si), where the laser fluence in the Gaussian beam dropped to a local value of *ϕ*_0_~0.22 J/cm^2^ (estimated by Equation (1)). Higher magnifications of the a-Si/c-Si interface are presented in Figure 6c,d, revealing a transition zone with an extent of ~3.9 nm (see Figure 6c) and no indications of crystal defects formed upon re-solidification, e.g., twins or stacking faults (see Figure 6d). Such a low roughness of the a-Si/c-Si interface justifies the previously made assumption of flat interfaces in the ellipsometry layer model discussed in Section 3.1. The different structural order is demonstrated by the two fast Fourier transforms shown as insets in Figure 6c. The two-dimensional Fourier transforms (2D-FTs) were attained from high-resolution TEM images of the a-Si and c-Si regions.

**Figure 7:**
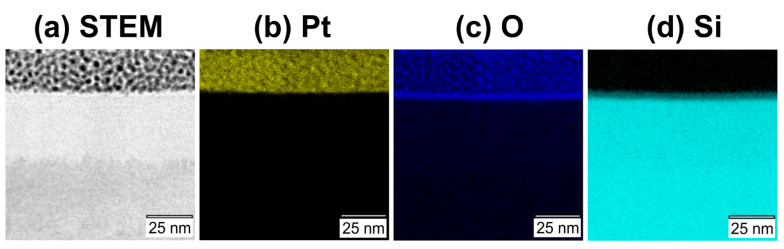
STEM image (**a**) and elemental energy dispersive X-ray spectroscopy (STEM-EDX) maps of platinum (Pt, yellow, (**b**)), oxygen (O, blue, (**c**)), silicon (Si, cyan, (**d**)).

Additionally, the lamella was investigated by STEM and EDX mapping for recording cross-sectional elemental maps of the chemical elements platinum (Pt), silicon (Si), and oxygen (O). Dopant elements were not detected due to their low concentration. In Figure 7, these elemental maps (512 × 512 pixels, Figure 7b–d) are compared to the corresponding STEM image as separate reference (Figure 7a).

**Figure 8:**
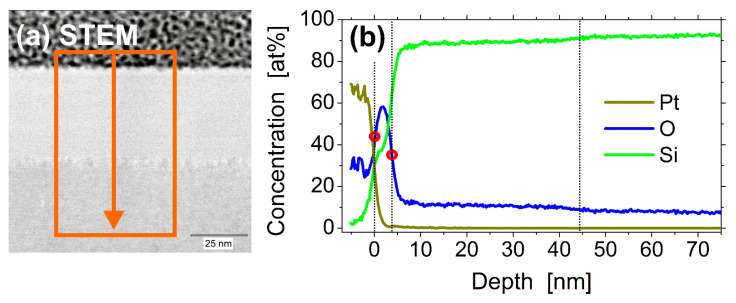
STEM image (**a**) and EDX-based depth profiles (**b**) of the relative atomic concentrations of platinum (Pt, olive green), oxygen (O, blue), silicon (Si, bright green). The data are obtained by binning over the region of interest (ROI) indicated in (**a**) as an orange rectangle. The red circles in (**b**) mark the half-maximum positions of the oxide layer interfaces. The vertical dotted lines indicate the nominal positions of the layer interfaces.

The elemental maps confirm the previously made assignment of the cross-sectional layer structure and demonstrate homogeneous distributions of the elements Pt (see Figure 7b), O (see Figure 7c), and Si (see Figure 7d) within their respective regions. For a complementary quantification of the layer thicknesses of the silicon oxide and the a-Si layers with an improved signal-to-noise ratio, the EDX mapping data were binned across a width of ~53 nm in the direction parallel to the layer interfaces (see Figure 8a). The corresponding accumulated EDX counts of Pt, O, and Si are plotted in Figure 8b versus the position in the *z*-direction (depth), with the origin (*z* = 0) located at the Pt–sample interface.

The Pt-signal of the capping layer exhibits a sharp transition to the underlying native oxide silicon layer. No significant mixing of Pt with the silicon oxide can be detected here. The concentration curve of the oxygen exhibits a peak at a depth of ~1.8 nm and further confirms the information obtained by the TEM imaging. For calculation of the thickness of the oxide layer, a 50% criterion was applied. Evaluating the difference of the half-maximum positions (marked by red circles) of the two interfaces of the oxide layer results in an averaged thickness of ~3.8 nm here (Figure 8b, blue curve). This value well agrees with the silicon oxide layer thickness of 3.5 nm obtained from Figure 5 in the SIE-based approach discussed in Section 3.1.

Interestingly, the oxygen signal does not completely vanish in regions associated with the silicon: within the amorphous silicon layer, a concentration around ~11 at% of O was detected over a depth of *d*_am_ ~44 nm, before saturating at a level of ~8 at% in the region of single-crystalline silicon (Figure 8b, blue curve). Different effects may contribute to this finding. First, during the period of time in the liquid state (melt duration) of the silicon (a few nanoseconds only for the given laser irradiation conditions [34]), some oxygen may dissolve in the liquid silicon and remain in the material after re-solidification. Second, after the preparation of the FIB lamella and during its storage and transfer to the TEM device, a thin native oxide layer will have formed at the two surfaces of the lamella, before being investigated in TEM/STEM. Local difference between the a-Si and c-Si regions may then be caused by the slightly different surface oxidation conditions at the altered silicon lattice structure. In view of the short melt duration and the rather homogeneous distribution of O across the entire depth of the a-Si layer, the second scenario appears to be more likely here.

## 4. Discussion

**Figure 9:**
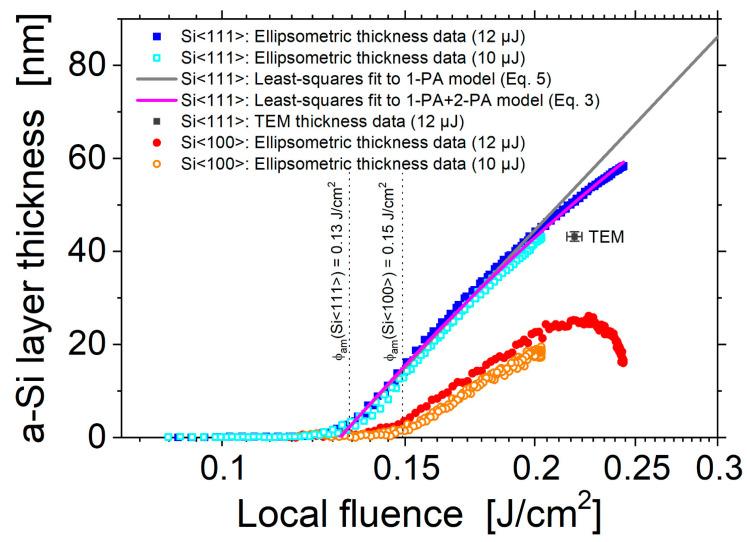
Amorphous layer thickness *d*_am_ versus local laser fluence *ϕ*. The data points are obtained from SIE measurements at the four different fs-laser-irradiated spots analyzed previously in Figure 4 for Si<111> (blue full squares (12 µJ) and cyan open squares (10 µJ)) and for Si<100> (red full circles (12 µJ) and open orange circles (10 µJ)). A quantitative measurement by TEM is indicated as a single black square with its respective uncertainties. The two vertical dashed lines indicate the corresponding amorphization thresholds of both silicon materials as previously deduced by OM. The solid pink and solid gray lines are least-squares fits to Equations (3) and (5), respectively.

In order to elucidate more details of the fs-laser-induced amorphization and particularly to study its fluence dependence, the data of the amorphous silicon layer obtained by SIE (Section 3.1) were further analyzed. On basis of Equation (1), for all four laser-irradiated spots displayed in Figure 4, each image pixel location (*x*,*y*) was associated with a local fluence value *ϕ*(*r* = √(*x*^2^ + *y*^2^)). Figure 9 semi-logarithmically re-plots the resulting amorphous silicon layer thickness profiles *d*_am_ as a function of the local laser fluence *ϕ* for horizontal cross-sections through the center of the spots displayed in a local fluence range between 0.08 and 0.3 J/cm^2^. For quantitative comparison, the thickness of the amorphous layer measured by TEM on Si<111> was added as an experimental data point (black full square) at its local fluence value calculated by Equation (1) from the position indicated in Figure 1. At that local fluence of *ϕ*_0_ ~0.22 J/cm^2^, the a-Si thickness value of ~50 nm predicted by the SIE curve deviates by ~10% to 15% from that quantified by TEM—a reasonably good agreement in view of the assumptions made in the SIE modeling and the complexity of the experiments involved. Supposedly, the deviation mainly arises from minor differences of the optical constants of the fs-laser-amorphized silicon and the covering silicon oxide to the literature values applied here for the SIE modeling [63] and from the small surface roughness that was not considered by the optical model.

Obvious differences can be seen between the data sets of the Si<111> and Si<100> wafer materials. Most strikingly, the amorphous layer thickness (slope) is significantly larger for Si<111>, and two different thresholds of *ϕ*_am_<111> = 0.13 J/cm^2^ and *ϕ*_am_<100> = 0.15 J/cm^2^ can be seen for the onset of superficial amorphization (compare the set of blue/cyan data points with the red/orange ones in Figure 9). Both findings (slopes, thresholds) reflect the reduced amorphization tendency of Si<100> arising from its larger critical interfacial velocity value (see the discussion in Section 3). Importantly, close to the amorphization threshold fluence, all curves exhibit a linear scaling in this data representation. This finding confirms a functional dependence in the form of *d*_am_~ln(*ϕ*/*ϕ*_am_) that was previously assumed already in [42]. Moreover, using that relation together with the radial fluence profile *ϕ* = *ϕ*(*x*,*y*) presented in Equation (1), it becomes clear that the amorphous layer thickness profiles shown in Figure 4a,c exhibit in a good approximation a parabolic thickness profile in the form *d*_am_(*x*,*y*)~[ln(*ϕ*_0_/*ϕ*_am_) − 2(*x*^2^+*y*^2^)/*w*_0_^2^] [42]. For local laser fluences exceeding ~0.2 J/cm^2^, some deviations from this simple scaling law of *d*_am_~ln(*ϕ*/*ϕ*_am_) are notable in Figure 9. While for the Si<111> a moderate thickness saturation occurs, for the Si<100> a thickness reduction can be seen in the fluence range between 0.22 and 0.24 J/cm^2^. At even larger fluences (*ϕ* > *ϕ*_cr_) re-crystallization sets in for the Si<100> (see Figure 3), resulting in *d*_am_~0 nm.

Some further conclusions can be drawn from a mathematical analysis of the absorption processes of the laser radiation occurring in the silicon upon ultrashort-pulse irradiation. For simplicity, the analysis is performed at the center of the laser spot, neglecting the Gaussian beam profile here. The absorption of the optical radiation at ~800 nm wavelength in crystalline silicon is promoted either via linear absorption (1-PA) processes (via indirect interband transitions or via free-carrier absorption (FCA) through already existent carriers in the conduction band) or via direct two-photon absorption (2-PA). Neglecting diffusion and recombination processes during the optical excitation, the intensity distribution *I*(*z*) of the laser beam propagating inside the silicon along the *z*-direction perpendicular to the surface can be described by a one-dimensional ordinary differential equation [25,57]:

(2) $$\frac{dI\left(z,t\right)}{dz}=-\left[\alpha_{0}+\alpha_{\mathrm{fca}}\left(z,t\right)+\beta \cdot I\left(z,t\right)\right]\cdot I\left(z,t\right)$$

α_0_ and *β* denote the linear and two-photon interband absorption coefficients, respectively. The (linear) intraband free-carrier absorption is included via coefficient α_fca_(*z*,*t*). Since the FCA depends linearly on the number of available (laser-induced) carriers in the conduction band, the spatial attenuation of the laser intensity with *z* is strongly affected by the transient electron density and can strongly change during the fs-laser pulse and with depth [35].

Given the depth- and time-dependence of α_fca_, in general, the differential equation must be solved numerically, which is beyond the scope of this work. However, it is instructive to analyze the limiting solutions of Equation (2); upon neglecting the FCA and assuming solely 1-PA and 2-PA, it can be solved analytically via separation of the variables along with the boundary condition that the intensity at the surface *I*(*z* = 0) = *I*_0_ is reduced to a level of *I*(*d*_m_) = *I*_th,m_ at the depth *d*_m_ where the melting threshold *I*_th,m_ is reached. The solution can then be re-written as the following equation for the melt depth when relating the intensity in the solid to the incident peak fluence (*I*_0_ = (1 − *R*)*ϕ*_0_/τ) [64].

(3) $$d_{m}=\frac{1}{\alpha_{0}}\cdot \ln\left(\frac{\left[\frac{\alpha_{0}}{\phi_{\mathrm{th},m}}\;+\;\frac{\beta \left(1\;-\;R\right)}{\tau }\right]\cdot \phi }{\alpha_{0}+\frac{\beta \left(1\;-\;R\right)}{\tau }\cdot \phi }\right)$$

with *R* being the surface reflectivity at the laser wavelength, τ the laser pulse duration, and *ϕ*_th,m_ the melting threshold (not to be confused with the amorphization threshold *ϕ*_am_ here). For dominating 2-PA (α_0_ = 0), the melt depth reads [65]

(4) $$d_{m}=\frac{\tau }{\beta \left(1-R\right)}\cdot \left(\frac{1}{\phi_{\mathrm{th},m}}-\frac{1}{\phi }\right)$$

while for dominating 1-PA (*β* = 0), the melt depth simplifies to

(5) $$d_{m}=\frac{1}{\alpha_{0}}\cdot \ln\left(\frac{\phi }{\phi_{\mathrm{th},m}}\right)$$

Note that for pure 2-PA at large fluences *d*_m_ saturates at a constant value (see Equation (4)). Least-squares fits were performed using the analytical solutions of Equations (3) and (5) to the experimental data for the irradiation of Si<111> at *E*_p_ = 12 µJ (*ϕ*_0_ = 0.24 J/cm^2^) presented in Figure 9. The surface reflectivity of crystalline silicon was assumed to be constant during the laser pulse duration of τ = 30 fs and accounts to *R* = 0.33 for 790 nm radiation at normal incidence [40]. α_0_ and *β* were chosen as free fit parameters, while the melting threshold was assumed to coincide with the amorphization threshold (*ϕ*_th,m_ = *ϕ*_am_). The values of α_0,fit_ = 4.38 × 10^4^ cm^−1^ and *β*_fit_ = 15.7 cm/GW obtained from the least-squares fits to Equation (3) strongly deviate from the literature values (α_0_~1.1 × 10^3^ cm^−1^ [40] and *β*~6.8 cm/GW [66]), indicating that the FCA makes a significant contribution to a reduction of the fs-laser-induced melt depth in our experiments and that Equation (3) cannot be applied here. This becomes also obvious when comparing the (small signal) optical penetration depth 1/α_0_(790 nm) = 9.95 µm with the value 1/α_0,fit_ = 0.10 µm deduced from the least-squares fit to Equation (5) that is deviating by two orders of magnitude here. Note that the latter value is consistent with the melt depths reported for silicon when solving the full Equation (2) numerically in combination with a carrier density rate equation, a two-temperature model (TTM) and molecular dynamics (MD) simulations for somewhat longer Ti:Sapphire pulses of τ = 130 fs [35] and the maximum melt depth values obtained in time-resolved experiments [67].

Nevertheless, another important conclusion can be drawn from the remarkably different slopes of the experimental data in Figure 9 observed for Si<111> and Si<100> at local fluences close to the amorphization thresholds. For Si<100> the slope value is reduced by a factor of ~2 compared to the Si<111>. Since the coupling of the optical pulse energy into the silicon is not expected to depend significantly on the lattice orientation (resulting in the same melt depth *d*_m_), it is inferred here that the approximately two-times-smaller amorphization layer thicknesses dam must arise from differences in the re-solidification process, caused by the fact that the (larger) critical velocity of amorphization is reached later for Si<100> compared to Si<111>. In other words, at least for the Si<100> the melt depth is significantly larger than the amorphous layer thickness [*d*_m_(Si<100>) >> *d*_am_(Si<100>)]. Nevertheless, as proven by the experimental data in Figure 9, a scaling law *d*_am_ = *d*_0_ × ln(*ϕ*/*ϕ*_am_) remains valid under these conditions, indicating that a constant fraction of the maximum melt depth solidifies as amorphous top-layer. In contrast, for the Si<111> no indications were found by TEM/STEM that the melt depth may be different from the amorphous layer thickness—although it cannot completely be ruled out here if a perfectly epitaxial re-growth occurs.

Table 1 compiles the different threshold fluences (*ϕ*_am_, *ϕ*_annu_, *ϕ*_abl_, *ϕ*_cr_) analyzed in this work along with a comparison to other relevant literature values.

## 5. Conclusions

Single near-infrared fs-pulse laser-induced amorphization and re-crystallization of single-crystalline silicon (<111> and <100>) were studied with unprecedented resolution in a multi-method approach involving top-view optical microscopy (OM), spectroscopic imaging ellipsometry (SIE), atomic force microscopy (AFM), cross-sectional-view high-resolution transmission electron microscopy (HRTEM), and imaging energy dispersive X-ray spectroscopy (EDX). For laser fluences below the ablation threshold in the melting regime, significant differences of the thickness of the final amorphous surface layer were found among the two wafer materials. A multi-spectral all-optical approach based on SIE was developed allowing to retrieve spatially resolved thickness profiles with nanometric precision in a non-destructive manner. While for a radially Gaussian laser beam irradiating Si<111> at fluences up to 0.2 J/cm^2^ some parabolic amorphous layer profiles with maximum thicknesses of ~50 nm were determined, the corresponding maximum layer thickness for Si<100> was about half of this value at the same local fluence. The fs-laser generated amorphous silicon layer exhibited a very low interfacial roughness amplitude (~4 nm) to the underlying single-crystalline <111> substrate material. Neither stacking faults nor any traces of twinning were found by high-resolution TEM. The superficial oxide layer was analyzed by high-resolution EDX chemical mapping and depth profiling and indicated a thickness of ~3.5 nm, in agreement with the SIE-based thickness analysis. Moreover, the spectroscopic imaging ellipsometry was capable of visualizing a subtle laser-induced oxide thickness increase of ~2 nm in the laser-amorphized regions here. Mathematical modeling of the melt layer thickness on the basis of several different absorption processes (1-PA, 2-PA, FCA) underlined the relevance of free-carrier absorption processes in the fs-laser-induced melting of silicon. Our results support a scenario that for Si<111> the fs-laser-induced melt layer transfers completely into amorphous material, while for Si<100> only an upper part of the melt pool finally turns amorphous at the end of the solidification process when a critical interfacial velocity is exceeded. Moreover, laser-fluence-dependent re-crystallization effects can manifest in the center of the irradiated spots, i.e., for peak fluences exceeding ~0.25 J/cm^2^ for Si<100> and 0.68 J/cm^2^ for Si<111>, respectively. Our results provide evidence that spectroscopic imaging ellipsometry is capable of a fast and precise characterization of nanometer-thick laser-induced structural and chemical surface modifications.

## Figures and Tables

**Table 1:** Characteristic threshold fluence for the irradiation of single-crystalline silicon in air or vacuum by single Ti:Sapphire fs-laser pulses (τ = 30 fs to 150 fs, λ = 780 nm to 800 nm, normal incidence); specific experimental conditions are indicated as footnotes. Abbreviations: AFM: atomic force microscopy, CSLM: confocal scanning laser microscopy, OM: optical microscopy, OES: optical emission spectroscopy, µ-RS: micro-Raman spectroscopy, SEM: scanning electron microscopy, SIE: spectroscopic imaging ellipsometry, TEM: transmission electron microscopy.

Threshold	J/cm^2^	Effect	Detection Methods	Reference
*ϕ*_am_ Si<111>	0.13 ^1^ 0.27 ^2^	Melting and amorphization	AFM, OM, SIE, TEM AFM, CSLM, OM, μ-RS	This work [40]
Si<100>	0.15 ^1^ ~0.15 ^2^		AFM, OM, SIE OM	This work [68]
*ϕ*_annu_ Si<111>	0.26 ^1^ 0.41 ^2^	Destruction of the native oxide layer	AFM, OM AFM, CSLM, OM, μ-RS	This work [40]
Si<100>	0.29 ^1^		AFM, OM	This work
*ϕ*_abl_ Si<111>	0.39 ^1^ 0.52 ^2^	Ablation	OM, AFM AFM, CSLM, OM, μ-RS	This work [40]
Si<100>	0.37 ^1^ ~0.3–0.4 ^2^ ~0.3 ^3^		AFM, OM AFM, OES AFM, SEM, TEM	This work [69,70] [68]
*ϕ*_cr_ Si<111>	0.68 ^1^ 0.58 ^2^	Re-crystallization	AFM, OM, SIE AFM, CSLM, OM, μ-RS	This work [40]
Si<100>	0.25 ^1^		AFM, OM, SIE	This work

Pulse durations: ^1^ 30 fs. ^2^ 150 fs. ^3^ 130 fs.

## Data Availability

The data presented in this study are available on request from the corresponding author.
